# Investigating the use of support in secondary school: the role of self-reliance and stigma towards help-seeking

**DOI:** 10.1080/09638237.2022.2069720

**Published:** 2022-05-03

**Authors:** L. Beukema, A. F. de Winter, E. L. Korevaar, J. Hofstra, S. A. Reijneveld

**Affiliations:** a Department of Health Sciences, University Medical Center Groningen, University of Groningen, Groningen, The Netherlands; b Department of Rehabilitation, Hanze University of Applied Sciences, Groningen, The Netherlands

**Keywords:** Adolescents, mental health, stigma, self-reliance, school support, psychosocial problems

## Abstract

**Purpose: **Adolescents are the least likely to seek help for their mental health problems. School may be an important route to improve early recognition of adolescents with mental health problems in need for support, but little is known about the barriers to school support.

**Materials and methods:** Data were collected in a longitudinal cohort study of Dutch adolescents (age 12–16) in secondary school (*n* = 956). We assessed the relation between level of psychosocial problems at the beginning of the school year (T1) and the support used in school at the end of that school year (T2), whether the willingness to talk to others (measured at T1) mediates this relation, and whether stigma towards help-seeking (T1) moderates this mediation.

**Results:** Adolescents with more psychosocial problems were more likely to use support in school and were less willing to talk to others about their problems, but the willingness to talk to others was not a mediator. Stigma moderated the relationship between psychosocial problems and willingness to talk to others.

**Conclusions:** Most adolescents with psychosocial problems get support in Dutch secondary school regardless of their willingness to talk to others about their problems. However, perceiving stigma towards help-seeking makes it less likely for someone to talk about their problems.

## Introduction

Worldwide, adolescents frequently do not seek help for their mental health problems (Michel et al., 2018); in fact, they are reportedly the least likely age group to seek help for their mental health problems (Polanczyk et al., 2015; Reavley et al., 2010; Rickwood et al., 2007). Estimates are that up to 50% of children and adolescents suffer from a mental disorder (at one point in their life) (Merikangas et al., 2010; Ormel et al., 2015) and several studies have shown that only 18–34% of adolescents with increased depressive or anxious symptoms seek professional help (Bland et al., 1997; Gulliver et al., 2010; Haavik et al., 2019). Mental health problems in adolescence are associated with lower educational attainment (Hjorth et al., 2016; Veldman et al., 2014), and poorer socio-economic outcomes later in life (Kieling et al., 2011). Support in school for adolescents with mental health problems is important as it might prevent school drop-out, improve educational attainment of adolescents, and improve later life outcomes (Suldo et al., 2011).

School may be an important route to improve early recognition of adolescents in need for support for their mental health problems (Magnusson & Låftman, 2019; Weare & Nind, 2011). Schools can relatively easily offer support services to adolescents in school, since adolescents spend a major part of their time in school and support staff is often already present (Arora et al., 2016). Support services, such as help with planning, organizing, attention, concentration, stress, school-absence, or classroom behavior, are available in most secondary education schools (in the Netherlands) and can be a potential way of supporting those with mental health problems, since mental health problems seem related to the various cognitive functions addressed in these support services (Brière et al., 2015; Demetriou et al., 2018; Knight & Baune, 2018; Sonuga-Barke et al., 2016; Suldo et al., 2011). However, to ensure the support services are also adequately accessible for adolescents with mental health problems, it is important to understand the factors that influence help-seeking and the consequential use of support in school, to mitigate the negative impact of mental health problems on school functioning. Several studies investigated help-seeking in the context of specific school-based interventions or specific disorders (Calear et al., 2022; Gijzen et al., 2018), but there is a lack of research on factors that influence adolescents in receiving or seeking support in school.

Several factors have been identified as potential barriers towards help-seeking. Gulliver and colleagues (2010) concluded from their systematic review that perceived stigma and embarrassment, difficulties recognizing symptoms, and a preference for self-reliance are the most important personal barriers to help-seeking in adolescents. Specifically, they found that they prefer to rely on themselves instead of seeking help for their problems (i.e., by not talking to others about one’s problems). Self-reliance, defined as reliance on one’s own efforts and abilities, has been identified in multiple other studies to play a prominent role in seeking help for mental health problems (Andrade et al., 2014; Sheppard et al., 2018). Therefore, adolescents with mental health problems might be at a lower chance of using support in school when they are not willing to talk to others about problems.

Multiple theories or frameworks have been used to explain help-seeking behavior in previous research. As mentioned above, stigma is widely regarded as a major factor with respect to help-seeking (Aguirre Velasco et al., 2020; Corrigan, 2004; Corrigan et al., 2014; Gulliver et al., 2010; Jung et al., 2017; Nearchou et al., 2018; Pedersen & Paves, 2014; The Lancet, 2016). The concept of stigma is complex and multifaceted, and can be defined and categorized differently based on the perspective used (e.g., who or what gives or receives the stigma) (Henderson & Gronholm, 2018). One way is to divide the concept of stigma in regard to mental health into public and self-stigma (Corrigan & Shapiro, 2010). Public stigma refers to an individual’s perception of the attitudes of the general public towards people with mental health problems and self-stigma refers to individuals’ stigma attitudes towards themselves (Rüsch et al., 2005; Schnyder et al., 2017). However, help-seeking stigma has been found to be distinguishable from public and self-stigma (Schnyder et al., 2017). In particular, help-seeking stigma includes the specific stigma attitudes associated with seeking help (i.e., perception of need for help, openness about problems, trust that the help offered is adequate). In the current study, we focus on perceived stigma attitudes towards help-seeking (further referred to as “help-seeking stigma”), which refers to one’s perception of the stigma attitudes towards help-seeking for mental health problems, and is found to be a major barrier to help-seeking among young adults (Pedersen & Paves, 2014). These stigma attitudes may influence getting support by potentially increasing preferences for self-reliance: when young adults expect to experience negative social consequences when being open about their problems or receiving support for their problems, they would rather handle the problem on their own (Jennings et al., 2015). This could present as being afraid to disclose or talk about their problems with others. These factors have been investigated separately in relation to help-seeking, but more complex pathways towards the use of support have been little investigated (Michel et al., 2018).

**Figure 1.**
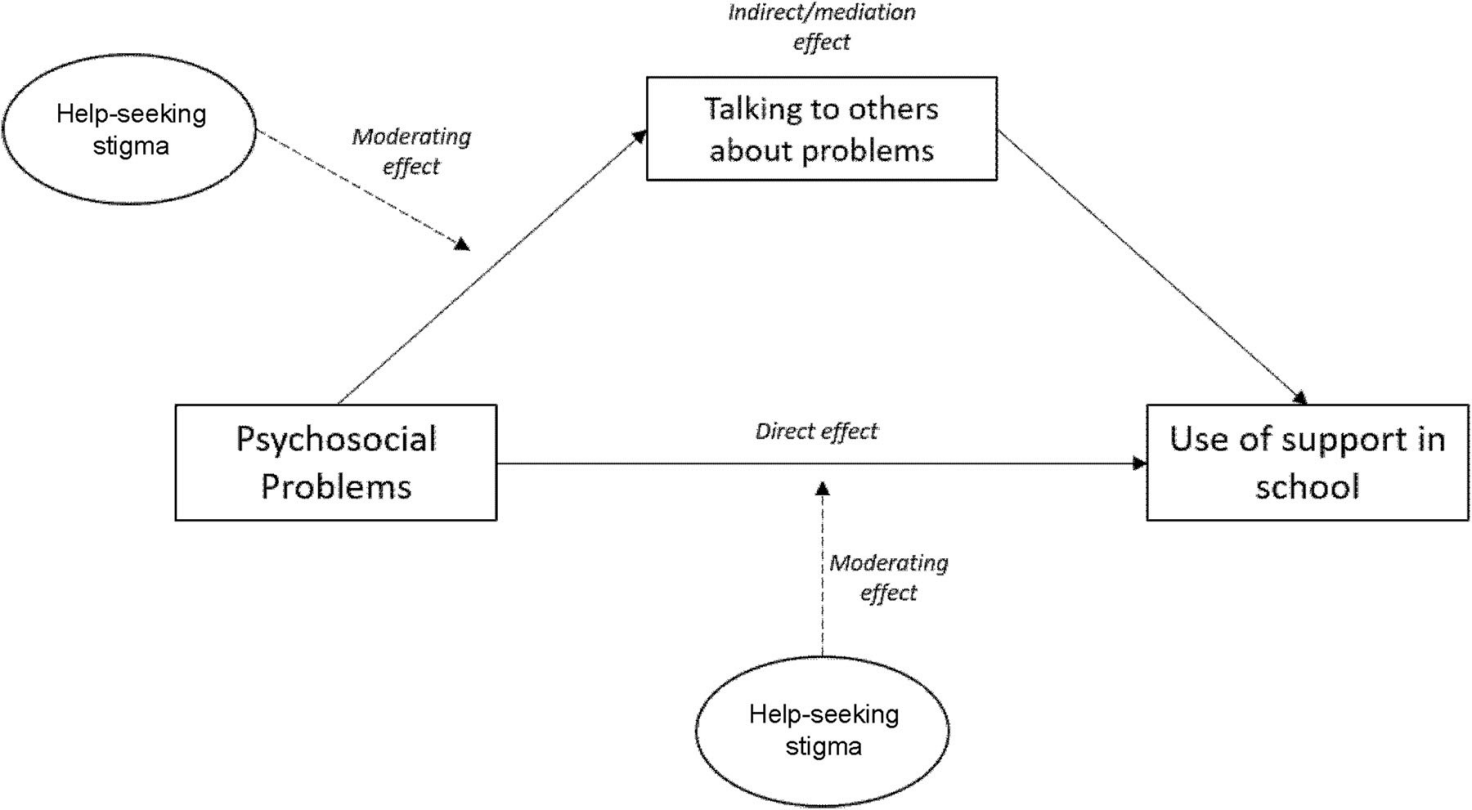
The hypothesized moderated mediation model.

In the current study, we hypothesize that adolescents with psychosocial problems are more likely to use support in school and that this association is mediated by their willingness to talk to others about their problems (see Figure 1). We furthermore hypothesize that help-seeking stigma moderates the adolescents’ willingness to talk to others about their problems. Finally, we hypothesize that the relation between psychosocial problems and use of support in school is moderated by the adolescents’ help-seeking stigma.

## Method

### Participants

Adolescents from grades 1 and 3 (ages 10–12 and 14–16) from ten secondary schools were invited to participate in an online survey at the beginning of the school year (T1, September 2017) and one at the end of the school year (T2, June 2018). The participating schools included all levels of regular secondary education: lower vocational education (VMBO-bb, VMBO-kb), and lower to higher secondary education levels (VMBO-tl, HAVO, VWO). For an overview of the Dutch educational system, we refer to Veldman et al. (Veldman et al., 2014). Special needs education or practical training schools were excluded, because all students in these schools receive extra support which does not fit with the goal of this study.

We used a passive informed consent procedure (in agreement with Dutch ethics protocol). This procedure involved the school sending information letters to the parents and adolescents four weeks before the survey. If the adolescent or one of the parents did not consent to participation, they were required to inform the school administration by e-mail or letter. Finally, the adolescent was asked to indicate their consent again at the start of the survey. If a parent and/or an adolescent did not consent to participate (either via the school administration or at the beginning of the survey), the adolescent was instructed to do homework in a separate classroom during the assessment.

From the ten participating schools, 2722 adolescents were invited to participate. Of these, 2648 (97%) filled in the first questionnaire. The second questionnaire was filled in by 1771 (65%) adolescents due to the fact that two schools were not able to participate at T2 because of organizational issues. The final sample included only those adolescents who we could identify that filled in both questionnaires, which lead to a sample of n = 956. To create this sample, we compared participants of both surveys based on the following identifiers: date of birth, zip-code, gender, and school level.

The study was performed in accordance with the Helsinki Declaration, and informed consent was obtained from all participants. The Medical Ethical Committee of the University Medical Centre Groningen deemed the study exempt from human subjects’ review (a non-WMO study).

### Procedure and measures

The adolescents filled in the survey at school during school hours on a computer, laptop, or their smartphone. During the assessment either a teacher, a researcher, or both were present in the classroom to provide assistance.

#### Dependent variable

Use of support was assessed at the end of the school year (T2) by asking the question: “Did you receive support in school in the past school year?”. The question was introduced with possible reasons for using support in school, such as: needing extra help in school, not doing well in school, or not feeling well in school. Answer options were “yes” or “no”. Support in school included any type of support for problems in school functioning due to emotional, behavioral or, social problems, such as help with planning, organizing, concentration, stress, school-absence, or classroom behavior.

#### Independent variable

Psychosocial problems were assessed at the start of the school year (T1) with the Dutch self-report version of the Strengths and Difficulties Questionnaire (SDQ) (Goodman, 2001; Muris et al., 2003; van Widenfelt et al., 2003). This self-report version has been shown to validly assess emotional and behavioral problems in adolescents (Theunissen et al., 2019; Vugteveen et al., 2021). The total difficulties score (TDS) was based on the past six months, and ranges from 0 to 40 with higher scores indicating more problems. The Dutch norm for a normal level of psychosocial problems in adolescents is a TDS below 11 (Vogels et al., 2009).

#### Mediating variable

Willingness to talk to others was measured at T1 by asking the question: “If you are not feeling great about yourself and school is not going well, with whom would you like to talk about it?”. Answer options were parents, friends, boyfriend/girlfriend, mentor at school, teacher at school, other family, or no-one. This was recoded to a dichotomous variable, with the answers “I would talk to someone” (1) and “I would talk to no-one” (0).

#### Moderating variable

Stigma attitudes were measured at T1 with the Stigma Scale for Receiving Psychological Help (SSRPH) (Komiya et al., 2000). This instrument measures perceived stigma of help-seeking. Five items are rated on a 4-point scale ranging from 0 (strongly disagree) to 3 (strongly agree). Total scores ranged from 0 to 15 with higher scores indicating greater help-seeking stigma. The SSRPH was translated into Dutch using back translation by six independent translators (three forward, three back). We pilot tested the translation among 500 adolescents (aged 12–16), leading to additional editing to match the language level of the age group under investigation and the context of the Dutch school environment.

#### Background variables

Participant characteristics comprised age, gender, and educational level. Age was dichotomized into two groups, ages 10–12 (0) and 14–16 (1), corresponding to the grade the participant was in (first or third grade). Gender was defined as either male (0), or female (1). Educational level was dichotomized into two groups, lower secondary education (VMBO) levels (1), and higher secondary education levels (HAVO/VWO) (0).

### Statistical analyses

We first described the background characteristics of the sample. Then we assessed the direct association between the level of psychosocial problems of adolescents and their use of support using logistic regression. After confirming this direct relation, we used structural equation modelling (SEM) to analyze our theory-based model (Figure 1) step by step. Our theory-based model is partly temporal: the predictor and the mediator were measured simultaneously (T1), however, the predictor (psychosocial problems) referred to the past 6 months, while the mediator referred to the moment of data-collection. Use of support was assessed at the end of the school year (T2).

The steps in our analysis were as follows: we first determined the association between level of psychosocial problems and use of support (Model 1). Second, we added talking to others to the model to assess whether talking to others mediated this relation (Model 2). Third, we added help-seeking stigma as an interaction term to the model to assess whether help-seeking stigma moderated the association between psychosocial problems and talking to others (Model 3). Finally, we assessed whether help-seeking stigma moderated the association between psychosocial problems and use of support (Model 4). We did so by building upon Model 3 and adding help-seeking stigma to the model as an interaction term. All models were estimated through Maximum Likelihood Estimation. Model fit was assessed by comparing the Akaike Information Criterion (AIC) and Bayesian Information Criterion (BIC) of every model to compare the model fit of the models in every step (West et al., 2012). We aimed to test our hypothesized model, and not select the best fitting model, making that our choice of the best model was based on both fit with theory and statistical. Unstandardized estimates are presented. Descriptive and logistic regression analyses were performed using SPSS (version 26) and SEM-analyses were performed using Mplus (version 8.4).

Missing data was not imputed and assumed to be completely random (MCAR), based on Little’s test (*X^2^*(4, *N* = 956) = 7.361, *p* = .118). As for the loss to follow up between T1 and T2: we used a One-way ANOVA to compare the characteristics (age, gender, ethnicity, educational level, and level of psychosocial problems) of the adolescents that filled in only T1, only T2, and those that filled in both T1 and T2, and we did not find any significant differences.

## Results

### Characteristics of the participants

Table 1 shows the characteristics of the sample (*n* = 956). The average age of the participants at T1 was 13.2 years (*SD* 1.2). In general, the level of help-seeking stigma was low (*M* = 3.5, *SD* = 2.6), and the mean level of psychosocial problems fell in the normal range (SDQ TDS < 11). Of the adolescents who did not use support in school (*n* = 710), 44% had a heightened or abnormal level of psychosocial problems. Of the adolescents who did use support, 51% had a heightened or abnormal level of psychosocial problems. The majority indicated that they would talk to someone if they had problems, and mainly to their parents or their friends (see Table 1).

**Table 1.** Descriptive characteristics of the total sample and per use of support.

	Total *n* = 926^b^	Support^a^*n* = 216	No support *n* = 710
Gender *n* (%)			
Female	519 (54%)	121 (56%)	383 (54%)
Male	437 (46%)	95 (44%)	327 (46%)
Ethnicity *n* (%) Dutch	725 (76%)	159 (74%)	543 (77%)
School grade *n* (%)			
First	441 (46%)	123 (57%)	299 (42%)
Third	515 (54%)	93 (43%)	411 (58%)
School level n (%)			
Lower secondary	578 (60%)	140 (64%)	424 (60%)
Intermediate/higher secondary	378 (40%)	76 (36%)	286 (40%)
Willingness to talk *n* (%)			
No	65 (7%)	9 (4%)	54 (8%)
Yes	860 (90%)	200 (93%)	632 (89%)
Parent	703 (74%)	154 (71%)	526 (74%)
Brother/Sister	183 (19%)	39 (18%)	139 (20%)
Other family	95 (10%)	22 (10%)	69 (10%)
Friend(s)	486 (51%)	129 (60%)	341 (48%)
Teacher/school mentor	288 (30%)	88 (41%)	186 (28%)
Psychosocial problems *M* (*SD*)	9.7 (4.9)	10.9 (4.7)	9.2 (4.8)
Stigma *M* (*SD*)	3.5 (2.6)	3.7 (2.7)	3.5 (2.6)

^a^Use of support during the school year was measured at T2. All other variables were measured at T1.

^b^Values not always add up due to missing values.

### Psychosocial problems and use of support

For all estimated parameters per model, see the table in Appendix A. The results from Model 1 show that psychosocial problems at the beginning of the school year were significantly related to the use of support during the school year (*OR* =* 1.07, 95% CI [1.04, 1.10]*). In addition, adolescents with more psychosocial problems reported a higher help-seeking stigma, more often had a lower educational level, and were older.

### Mediating effects

When adding the mediator talking to others to the model the results show that a higher level of psychosocial problems was significantly associated with less willingness to talk to others (*OR* =* 0.90, 95%CI [0.86, 0.95]*) and more willingness to talk to others was associated with more use of support (*OR = 2.36, 95%CI [1.13, 4.92]*) (Model 2).

### Moderating effects

**Figure 2.**
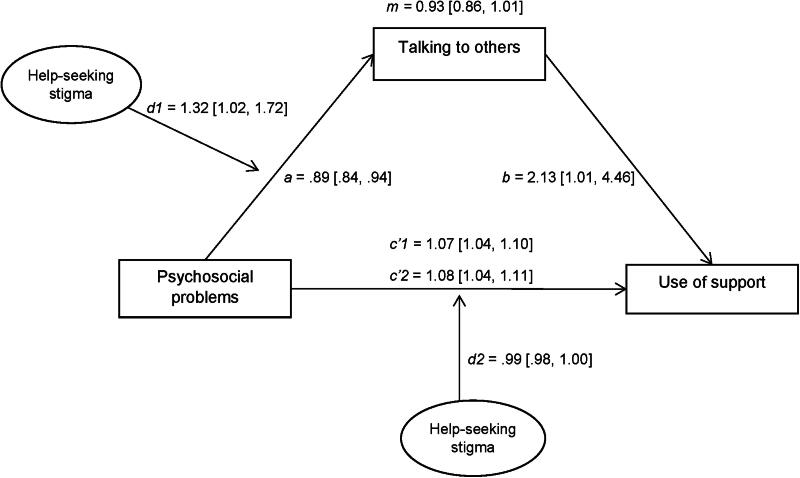
Results of moderated mediation analysis of talking to others and stigma: direct and indirect effects. Estimates are unstandardized odds-ratios (OR [95% CI]). All estimates are adjusted for age, gender, and educational level. a: direct association between psychosocial problems and talking to others; b: direct association between talking to others and use of support; c’1: direct association between psychosocial problems and use of support; c’2: direct association between psychosocial problems and use and support adjusted for mediator (talking to others) and moderator (perceived stigma); m: total indirect effect of talking to others; d1: moderating or interaction effect of stigma*psychosocial problems on talking to others; d2: moderating or interaction effect of stigma*psychosocial problems on use of support.

Stigma was a moderator of the relation between psychosocial problems and talking to others (*OR* =* 1.32, 95%CI [1.02, 1.72]*), however not a moderator of the relation between psychosocial problems and use of support in school (*OR* =* 1.00, 95%CI [1.00, 1.01]*) (see Figure 2).

## Discussion

The current study aimed to assess whether the willingness to talk to others mediated the relation between level of psychosocial problems and use of support at school, and whether help-seeking stigma moderated the mediation. We found that adolescents with a higher level of psychosocial problems are also more likely to use support for school functioning in secondary school. This association was not mediated by willingness to talk to others about their problems. In addition, help-seeking stigma did not moderate the relation between psychosocial problems and use of support. Stigma did moderate the relation between psychosocial problems and talking to others.

The majority of our sample reported that they would talk to someone about their problems, but prefer to talk to their parents or friends, which is consistent with other studies (Gulliver et al., 2010; Rickwood et al., 2007). However, there was a negative relation between the level of psychosocial problems and talking about problems: those with more problems were less willing to talk about problems with others. Previous research showed that a lack of willingness to talk to others, especially to professionals, inhibits help-seeking (Rickwood et al., 2005). Contrary to these findings, we found that in our sample most adolescents with increased psychosocial problems get support in school regardless of their willingness to talk about their problems. It is possible that in our sample, pupils with psychosocial problems receive support in school, because of their externalizing behavior, decreased participation in class, or changes in school functioning (Boer & Kuijper, 2020). In these cases, not being willing to talk to others about problems might not play a big role in the pathway to support in Dutch secondary school. Even though there are plenty of signs that supporting adolescents with mental health problems in secondary school remains difficult. However, there might be other factors than willingness to talk about problems that are more strongly associated with getting support, such as the adolescents’ ability to recognize their own problems and, consequently, their own perceived need for support.

Yet a considerable group of adolescents in our sample did not receive support in school, even though they reported heightened psychosocial problems. It is possible that their psychosocial problems do not affect their school functioning, and they therefore do not receive support in school. However, they may not be identified by school staff as needing support. Especially anxious youth often go undetected and untreated since their problems are less easily identified by their behavior (Essau, 2005). Moreover, educators are often not trained to address mental health problems, and lack necessary skills and strategies (O’Reilly et al., 2018; Reinke et al., 2011). It is also possible these adolescents already receive support outside of school for their psychosocial problems. Finally, another explanation is that these adolescents may not recognize their own need for support, or do not have confidence in the support available (Radez et al., 2020). It is important to consider the possibility that there are adolescents in secondary school with psychosocial problems that have an unmet need for support. Previous studies have shown that adolescents with high psychological distress and unmet needs for support are less likely to solve problems by themselves, use less informal help from friends or family, and report lower future help-seeking intentions (Sheppard et al., 2018).

We found that help-seeking stigma was not a moderator of the relation between psychosocial problems and the use of support, but was a moderator of the relation between the level of psychosocial problems and talking about problems, i.e., stigma strengthens the negative relation between psychosocial problems and talking to others. This can be interpreted as that perceiving a negative judgement about seeking help for a mental illness makes it less likely for someone to talk about her/his problems. This is in line with some previous research, which is often focused on the concept of disclosure concerns. This concept is defined as worrying about the decision to disclose a mental health condition to others, which was found to be a major stigma-related barrier to help-seeking (Clement et al., 2015; Mulfinger et al., 2019). Other studies identified perceived public stigma and personal attitudes towards mental illness or help-seeking as major factors in the use of support for mental health problems (Nearchou et al., 2018; Schnyder et al., 2017). This might explain why the findings in our study, in which stigma was defined and measured differently, deviates from this literature. However, as noted before, in Dutch secondary education adolescents with psychosocial problems are often identified by a school staff by their behavior, lack of participation, or their changed functioning in school. In that case, the level of help-seeking stigma perceived by an adolescent will not play a role in them getting support in school.

### Strengths and limitations

One important strength of this study is our large, longitudinal sample that includes adolescents from all educational levels. Second, we translated and adjusted the stigma questionnaire through a pilot (*n* = 500) to adequately tailor the questionnaire to our sample of young adolescents. Third, we investigated the role of stigma in help-seeking for mental health in a less investigated group: adolescents outside of the United States (Shechtman et al., 2018).

Our study also had some limitations. Firstly, the adolescents with the most severe problems might not have been present in school during the survey as they are more likely to be absent from school (Eekelen et al., 2010). Second, we used a broad definition of the term “support in school”. This is not without reason: the support offered in secondary school is mostly focused on problems in school functioning, also for those with mental health problems. Third, we did not measure whether the adolescents received support outside of school. It is possible that if they receive external support or services there may not be a need for school support, despite their mental health difficulties. Fourth, the simultaneous measurement of the predictor and mediators limits the potential to determine the direction of the associations between them.

### Implications and future research

The way in which talking to others and stigma play a role in the use of support in secondary school was different in our sample than we hypothesized. For future research we suggest the following. First, there are a number of factors suggested to play a role in the process of help-seeking. In the current study we looked at several individual factors, yet there are also structural factors (e.g., access to support) that could play a role (Radez et al., 2020). For example, it is likely that the way in which support in secondary schools is organized influences the use of support. School policies, regarding the type of support that is offered and to whom support is offered, differ per school. Thus, to understand the complex help-seeking process future research should also include these structural factors.

Second, in the current study we focused on perceived help-seeking stigma, while there is a differentiation between several subtypes of stigma, including self-stigma (stigma of a person towards themselves or internalized stigma). Self-stigma also seems to increase self-reliance and to delay help-seeking (Corrigan & Rao, 2012). Future research focused on help-seeking should include both types of stigmas to give a complete understanding of the role of stigma in help-seeking.

Third, our sample is taken from regular education schools, therefore our results are only generalizable to this population. Future research could also take into account special needs educations schools, as the relationships studied might be differently associated in that context.

Finally, as described in the limitations, our mediation model was partly based on simultaneous measurements, which limited the potential to determine the direction of the associations between the predictor and mediators. Our findings should therefore be confirmed by research with differently timed assessments for each variable in such a mediation model.

## Conclusion

In conclusion, there is room for improvement concerning adolescents’ use of support in school, especially in regard to the relation between adolescents’ willingness to talk to others about their problems and the role of stigma. Mental health education programs addressing stigma and help-seeking have the potential to promote openness about mental health, reduce stigma, and stimulate help-seeking in adolescents (Gronholm et al., 2017; Salerno, 2016; Wei et al., 2013), and as previous research showed, adolescents are more likely to seek help when they have the knowledge, skills, resources, and confidence to seek help. In addition, the implementation of mental health education programs in secondary schools can facilitate early identification of mental health problems and may even contribute to the prevention or decrease of mental health issues. However, there is still a lack of consensus on the effectiveness of such programs and on how to implement them (Feiss et al., 2019; Pinto-Foltz et al., 2011; Wei et al., 2013). Further research, especially using RCT and longitudinal designs, is needed, as it remains important to facilitate an appropriate environment in which adolescents with mental health problems can receive support.

## Data Availability

The data that support the findings of this study are available on request.

## References

[CIT0001] Aguirre Velasco, A., Cruz, I. S. S., Billings, J., Jimenez, M., & Rowe, S. (2020). What are the barriers, facilitators and interventions targeting help-seeking behaviours for common mental health problems in adolescents? A systematic review. *BMC Psychiatry*, *20*(1), 293. 10.1186/s12888-020-02659-0 32527236 PMC7291482

[CIT0002] Andrade, L. H., Alonso, J., Mneimneh, Z., Wells, J. E., Al-Hamzawi, A., Borges, G., Bromet, E., Bruffaerts, R., de Girolamo, G., de Graaf, R., Florescu, S., Gureje, O., Hinkov, H. R., Hu, C., Huang, Y., Hwang, I., Jin, R., Karam, E. G., Kovess-Masfety, V., … Kessler, R. C. (2014). Barriers to mental health treatment: Results from the WHO World Mental Health surveys. *Psychological Medicine*, *44*(6), 1303–1317. 10.1017/S0033291713001943 23931656 PMC4100460

[CIT0003] Arora, P. G., Connors, E. H., George, M. W., Lyon, A. R., Wolk, C. B., & Weist, M. D. (2016). Advancing evidence-based assessment in school mental health: Key priorities for an applied research agenda. *Clinical Child and Family Psychology Review*, *19*(4), 271–284. 10.1007/s10567-016-0217-y 27730441

[CIT0004] Bland, R. C., Newman, S. C., & Orn, H. (1997). Help-seeking for psychiatric disorders. *The Canadian Journal of Psychiatry*, *42*(9), 935–942. 10.1177/070674379704200904 9429063

[CIT0005] Boer, A. D., & Kuijper, S. (2020). Students’ voices about the extra educational support they receive in regular education. *European Journal of Special Needs Education*, *36*, 625–641. 10.1080/08856257.2020.1790884

[CIT0006] Brière, F. N., Janosz, M., Fallu, J.-S., & Morizot, J. (2015). Adolescent trajectories of depressive symptoms: Codevelopment of behavioral and academic problems. *The Journal of Adolescent Health : Official Publication of the Society for Adolescent Medicine*, *57*(3), 313–319. 10.1016/j.jadohealth.2015.05.012 26299558

[CIT0007] Calear, A. L., McCallum, S. M., Christensen, H., Mackinnon, A. J., Nicolopoulos, A., Brewer, J. L., Werner-Seidler, A., Morse, A. R., Kazan, D., Farrer, L. M., Kampel, L., & Batterham, P. J. (2022). The Sources of Strength Australia project: A cluster randomised controlled trial of a peer-connectedness school-based program to promote help-seeking in adolescents. *Journal of Affective Disorders*, *299*, 435–443. 10.1016/j.jad.2021.12.043 34952104

[CIT0008] Clement, S., Schauman, O., Graham, T., Maggioni, F., Evans-Lacko, S., Bezborodovs, N., Morgan, C., Rüsch, N., Brown, J. S. L., & Thornicroft, G. (2015). What is the impact of mental health-related stigma on help-seeking? A systematic review of quantitative and qualitative studies. *Psychological Medicine*, *45*(1), 11–27. 10.1017/S0033291714000129 24569086

[CIT0009] Corrigan, P. W. (2004). How stigma interferes with mental health care. *The American Psychologist*, *59*(7), 614–625. 10.1037/0003-066X.59.7.614 15491256

[CIT0010] Corrigan, P. W., Druss, B. G., & Perlick, D. A. (2014). The impact of mental illness stigma on seeking and participating in mental health care. *Psychological Science in the Public Interest : A Journal of the American Psychological Society*, *15*(2), 37–70. 10.1177/1529100614531398 26171956

[CIT0011] Corrigan, P. W., & Rao, D. (2012). On the self-stigma of mental illness: Stages, disclosure, and strategies for change. *Canadian Journal of Psychiatry. Revue canadienne de psychiatrie*, *57*(8), 464–469. 10.1177/070674371205700804 22854028 PMC3610943

[CIT0012] Corrigan, P. W., & Shapiro, J. R. (2010). Measuring the Impact of programs that challenge the public stigma of mental illness. *Clinical Psychology Review*, *30*, 907–922. 10.1016/j.cpr.2010.06.004 20674114 PMC2952670

[CIT0013] Demetriou, E. A., Lampit, A., Quintana, D. S., Naismith, S. L., Song, Y. J. C., Pye, J. E., Hickie, I., & Guastella, A. J. (2018). Autism spectrum disorders: A meta-analysis of executive function. *Molecular Psychiatry*, *23*(5), 1198–1204. 10.1038/mp.2017.75 28439105 PMC5984099

[CIT0014] Eekelen, J., Lanser, H., Schoenmakers, P., Schouten, M., van Rijbroek, C., Kwant, D., van Helvoirt, C., van Kessel, B., & Gadella, J. (2010). *Bevindingen dossieronderzoek thuiszitters 2010: thuiszitters, sneller terug naar school. [Results of school-absence investigation 2010: getting them back to school]*. www.ingrado.nl

[CIT0015] Essau, C. A. (2005). Frequency and patterns of mental health services utilization among adolescents with anxiety and depressive disorders. *Depression and Anxiety*, *22*(3), 130–137. 10.1002/da.20115 16175563

[CIT0016] Feiss, R., Dolinger, S. B., Merritt, M., Reiche, E., Martin, K., Yanes, J. A., Thomas, C. M., & Pangelinan, M. (2019). A systematic review and meta-analysis of school-based stress, anxiety, and depression prevention programs for adolescents. *Journal of Youth and Adolescence*, *48*(9), 1668–1685. 10.1007/s10964-019-01085-0 31346924 PMC7548227

[CIT0017] Gijzen, M. W. M., Creemers, D. H. M., Rasing, S. P. A., Smit, F., & Engels, R. C. M. E. (2018). Evaluation of a multimodal school-based depression and suicide prevention program among Dutch adolescents: Design of a cluster-randomized controlled trial. *BMC Psychiatry*, *18*(1), 124. 10.1186/s12888-018-1710-2 29747618 PMC5946540

[CIT0018] Goodman, R. (2001). Psychometric properties of the strengths and difficulties questionnaire. *Journal of the American Academy of Child and Adolescent Psychiatry*, *40*(11), 1337–1345. 10.1097/00004583-200111000-00015 11699809

[CIT0019] Gronholm, P. C., Henderson, C., Deb, T., & Thornicroft, G. (2017). Interventions to reduce discrimination and stigma: The state of the art. *Social Psychiatry and Psychiatric Epidemiology*, *52*(3), 249–258. 10.1007/s00127-017-1341-9 28144713 PMC5344948

[CIT0020] Gulliver, A., Griffiths, K. M., & Christensen, H. (2010). Perceived barriers and facilitators to mental health help-seeking in young people: A systematic review. *BMC Psychiatry*, *10*(1), 113. 10.1186/1471-244X-10-113 21192795 PMC3022639

[CIT0021] Haavik, L., Joa, I., Hatloy, K., Stain, H. J., & Langeveld, J. (2019). Help seeking for mental health problems in an adolescent population: The effect of gender. *Journal of Mental Health (Abingdon, England)*, *28*(5), 467–474. 10.1080/09638237.2017.1340630 28719230

[CIT0022] Henderson, C., & Gronholm, P. C. (2018). Mental health related stigma as a ‘wicked problem’: The need to address stigma and consider the consequences. *International Journal of Environmental Research and Public Health*, *15*(6), 1158. 10.3390/ijerph15061158 29865225 PMC6024896

[CIT0023] Hjorth, C. F., Bilgrav, L., Frandsen, L. S., Overgaard, C., Torp-Pedersen, C., Nielsen, B., & Bøggild, H. (2016). Mental health and school dropout across educational levels and genders: A 4.8-year follow-up study. *BMC Public Health*, *16*(1), 976. 10.1186/s12889-016-3622-8 27627885 PMC5024430

[CIT0024] Jennings, K. S., Cheung, J. H., Britt, T. W., Goguen, K. N., Jeffirs, S. M., Peasley, A. L., & Lee, A. C. (2015). How are perceived stigma, self-stigma, and self-reliance related to treatment-seeking? A three-path model. *Psychiatric Rehabilitation Journal*, *38*(2), 109–116. 10.1037/prj0000138 25844914

[CIT0025] Jung, H., von Sternberg, K., & Davis, K. (2017). The impact of mental health literacy, stigma, and social support on attitudes toward mental health help-seeking. *International Journal of Mental Health Promotion*, *19*(5), 252–267. 10.1080/14623730.2017.1345687

[CIT0026] Kieling, C., Baker-Henningham, H., Belfer, M., Conti, G., Ertem, I., Omigbodun, O., Rohde, L. A., Srinath, S., Ulkuer, N., & Rahman, A. (2011). Child and adolescent mental health worldwide: Evidence for action. *The Lancet*, *378*(9801), 1515–1525. 10.1016/S0140-6736(11)60827-1 22008427

[CIT0027] Knight, M. J., & Baune, B. T. (2018). Cognitive dysfunction in major depressive disorder. *Current Opinion in Psychiatry*, *31*(1), 26–31. 10.1097/YCO.0000000000000378 29076892

[CIT0028] Komiya, N., Good, G. E., & Sherrod, N. B. (2000). Emotional openness as a predictor of college students’ attitudes toward seeking psychological help. *Journal of Counseling Psychology*, *47*(1), 138–143. 10.1037/0022-0167.47.1.138

[CIT0029] Magnusson, C., & Låftman, S. B. (2019). Self-reported mental health problems in adolescence and occupational prestige in young adulthood: A 10-year follow-up study. *Children and Youth Services Review*, *101*, 174–180. 10.1016/j.childyouth.2019.04.006

[CIT0030] Merikangas, K. R., He, J. P., Burstein, M., Swanson, S. A., Avenevoli, S., Cui, L., Benjet, C., Georgiades, K., & Swendsen, J. (2010). Lifetime prevalence of mental disorders in U.S. adolescents: Results from the national comorbidity survey replication-adolescent supplement (NCS-A). *Journal of the American Academy of Child and Adolescent Psychiatry*, *49*(10), 980–989. 10.1016/j.jaac.2010.05.017 20855043 PMC2946114

[CIT0031] Michel, C., Schnyder, N., Schmidt, S. J., Groth, N., Schimmelmann, B. G., & Schultze-Lutter, F. (2018). Functioning mediates help-seeking for mental problems in the general population. *European Psychiatry : The Journal of the Association of European Psychiatrists*, *54*, 1–9. 10.1016/j.eurpsy.2018.06.009 30031990

[CIT0032] Mulfinger, N., Rüsch, N., Bayha, P., Müller, S., Böge, I., Sakar, V., & Krumm, S. (2019). Secrecy versus disclosure of mental illness among adolescents: I. The perspective of adolescents with mental illness. *Journal of Mental Health (Abingdon, England)*, *28*(3), 296–303. 10.1080/09638237.2018.1487535 30596301

[CIT0033] Muris, P., Meesters, C., & van den Berg, F. (2003). The Strengths and Difficulties Questionnaire (SDQ)-further evidence for its reliability and validity in a community sample of Dutch children and adolescents. *European Child & Adolescent Psychiatry*, *12*(1), 1–8. 10.1007/s00787-003-0298-2 12601558

[CIT0034] Nearchou, F. A., Bird, N., Costello, A., Duggan, S., Gilroy, J., Long, R., McHugh, L., & Hennessy, E. (2018). Personal and perceived public mental-health stigma as predictors of help-seeking intentions in adolescents. *Journal of Adolescence*, *66*(1), 83–90. 10.1016/j.adolescence.2018.05.003 29800758

[CIT0035] O’Reilly, M., Svirydzenka, N., Adams, S., & Dogra, N. (2018). Review of mental health promotion interventions in schools. *Social Psychiatry and Psychiatric Epidemiology*, *53*(7), 647–662. 10.1007/s00127-018-1530-1 29752493 PMC6003977

[CIT0036] Ormel, J., Raven, D., van Oort, F., Hartman, C. A., Reijneveld, S. A., Veenstra, R., Vollebergh, W. A. M., Buitelaar, J., Verhulst, F. C., & Oldehinkel, A. J. (2015). Mental health in Dutch adolescents: A TRAILS report on prevalence, severity, age of onset, continuity and co-morbidity of DSM disorders. *Psychological Medicine*, *45*(2), 345–360. 10.1017/S0033291714001469 25066533

[CIT0037] Pedersen, E. R., & Paves, A. P. (2014). Comparing perceived public stigma and personal stigma of mental health treatment seeking in a young adult sample. *Psychiatry Research*, *219*(1), 143–150. 10.1016/j.psychres.2014.05.017 24889842 PMC4086709

[CIT0038] Pinto-Foltz, M. D., Logsdon, M. C., & Myers, J. A. (2011). Feasibility, acceptability, and initial efficacy of a knowledge-contact program to reduce mental illness stigma and improve mental health literacy in adolescents. *Social Science & Medicine (1982)*, *72*(12), 2011–2019. 10.1016/j.socscimed.2011.04.006 21624729 PMC3117936

[CIT0039] Polanczyk, G. v., Salum, G. A., Sugaya, L. S., Caye, A., & Rohde, L. A. (2015). Annual Research Review: A meta-analysis of the worldwide prevalence of mental disorders in children and adolescents. *Journal of Child Psychology and Psychiatry, and Allied Disciplines*, *56*(3), 345–365. 10.1111/jcpp.12381 25649325

[CIT0040] Radez, J., Reardon, T., Creswell, C., Lawrence, P. J., Evdoka-Burton, G., & Waite, P. (2020). Why do children and adolescents (not) seek and access professional help for their mental health problems? A systematic review of quantitative and qualitative studies. *European Child & Adolescent Psychiatry*, *1*, 3. 10.1007/s00787-019-01469-4 PMC7932953 31965309

[CIT0041] Reavley, N. J., Cvetkovski, S., Jorm, A. F., & Lubman, D. I. (2010). Help-seeking for substance use, anxiety and affective disorders among young people: Results from the 2007 Australian national survey of mental health and wellbeing. *The Australian and New Zealand Journal of Psychiatry*, *44*(8), 729–735. 10.3109/00048671003705458 20636194

[CIT0042] Reinke, W. M., Stormont, M., Herman, K. C., Puri, R., & Goel, N. (2011). Supporting children’s mental health in schools: Teacher perceptions of needs, roles, and barriers. *School Psychology Quarterly*, *26*(1), 1–13. 10.1037/a0022714

[CIT0043] Rickwood, D. J., Deane, F. P., & Wilson, C. J. (2007). When and how do young people seek professional help for mental health problems? *Medical Journal of Australia*, *187*(S7), 35–39. 10.5694/j.1326-5377.2007.tb01334.x 17908023

[CIT0044] Rickwood, D. J., Deane, F. P., Wilson, C. J., & Ciarrochi, J. (2005). Young people’s help-seeking for mental health problems. *Australian e-Journal for the Advancement of Mental Health*, *4*(3), 218–251. 10.5172/jamh.4.3.218

[CIT0045] Rüsch, N., Angermeyer, M. C., & Corrigan, P. W. (2005). Mental illness stigma: Concepts, consequences, and initiatives to reduce stigma. *European Psychiatry : The Journal of the Association of European Psychiatrists*, *20*(8), 529–539. 10.1016/j.eurpsy.2005.04.004 16171984

[CIT0046] Salerno, J. P. (2016). Effectiveness of universal school-based mental health awareness programs among youth in the United States: A systematic review. *The Journal of School Health*, *86*(12), 922–931. 10.1111/josh.12461 27866385 PMC5123790

[CIT0047] Schnyder, N., Panczak, R., Groth, N., & Schultze-Lutter, F. (2017). Association between mental health-related stigma and active help-seeking: Systematic review and meta-analysis. *The British Journal of Psychiatry : The Journal of Mental Science*, *210*(4), 261–268. 10.1192/bjp.bp.116.189464 28153928

[CIT0048] Shechtman, Z., Vogel, D. L., Strass, H. A., & Heath, P. J. (2018). Stigma in help-seeking: the case of adolescents. *British Journal of Guidance & Counselling*, *46*(1), 104–119. 10.1080/03069885.2016.1255717

[CIT0049] Sheppard, R., Deane, F. P., & Ciarrochi, J. (2018). Unmet need for professional mental health care among adolescents with high psychological distress. *The Australian and New Zealand Journal of Psychiatry*, *52*(1), 59–67. 10.1177/0004867417707818 28486819

[CIT0050] Sonuga-Barke, E. J. S., Cortese, S., Fairchild, G., & Stringaris, A. (2016). Annual Research Review: Transdiagnostic neuroscience of child and adolescent mental disorders-differentiating decision making in attention-deficit/hyperactivity disorder, conduct disorder, depression, and anxiety. *Journal of Child Psychology and Psychiatry, and Allied Disciplines*, *57*(3), 321–349. 10.1111/jcpp.12496 26705858 PMC4762324

[CIT0051] Suldo, S., Thalji, A., & Ferron, J. (2011). Longitudinal academic outcomes predicted by early adolescents’ subjective well-being, psychopathology, and mental health status yielded from a dual factor model. *The Journal of Positive Psychology*, *6*(1), 17–30. 10.1080/17439760.2010.536774

[CIT0052] The Lancet (2016). The health crisis of mental health stigma. *In the Lancet*, *387*(10023), 1027. 10.1016/S0140-6736(16)00687-5 27025171

[CIT0053] Theunissen, M. H. C., de Wolff, M. S., & Reijneveld, S. A. (2019). The strengths and difficulties questionnaire self-report: A valid instrument for the identification of emotional and behavioral problems. *Academic Pediatrics*, *19*(4), 471–476. 10.1016/j.acap.2018.12.008 30639760

[CIT0054] van Widenfelt, B. M., Goedhart, A. W., Treffers, P. D. A., & Goodman, R. (2003). Dutch version of the Strengths and Difficulties Questionnaire (SDQ). *European Child & Adolescent Psychiatry*, *12*(6), 281–289. 10.1007/s00787-003-0341-3 14689260

[CIT0055] Veldman, K., Bültmann, U., Stewart, R. E., Ormel, J., Verhulst, F. C., & Reijneveld, S. A. (2014). Mental health problems and educational attainment in adolescence: 9-year follow-up of the TRAILS study. *PloS One*, *9*(7), e101751. 10.1371/journal.pone.0101751 25047692 PMC4105412

[CIT0056] Vogels, A. G., Crone, M. R., Hoekstra, F., & Reijneveld, S. A. (2009). Comparing three short questionnaires to detect psychosocial dysfunction among primary school children: A randomized method. *BMC Public Health*, *9*(489), 489. 10.1186/1471-2458-9-489 20035636 PMC2804619

[CIT0057] Vugteveen, J., de Bildt, A., Theunissen, M., Reijneveld, M., & Timmerman, M. (2021). Validity aspects of the strengths and difficulties questionnaire (SDQ) adolescent self-report and parent-report versions among Dutch adolescents. *Assessment*, *28*(2), 601–616. 10.1177/1073191119858416 31257902 PMC7883005

[CIT0058] Weare, K., & Nind, M. (2011). Mental health promotion and problem prevention in schools: What does the evidence say? *Health Promotion International*, *26*(suppl 1), i29–i69. 10.1093/heapro/dar075 22079935

[CIT0059] Wei, Y., Hayden, J. A., Kutcher, S., Zygmunt, A., & McGrath, P. (2013). The effectiveness of school mental health literacy programs to address knowledge, attitudes and help seeking among youth. *Early Intervention in Psychiatry*, *7*(2), 109–121. 10.1111/eip.12010 23343220

[CIT0060] West, S., Taylor, G., Aaron, B., & Wu, W. (2012). Model fit and model selection in Structural Equation modeling. In R. Hoyle (Ed.), *Handbook of structural equation modeling* (pp. 209–231). The Guilford Press.

